# Height Uniformity Simulation and Experimental Study of Electroplating Gold Bump for 2.5D/3D Integrated Packaging

**DOI:** 10.3390/mi13091537

**Published:** 2022-09-17

**Authors:** Wenchao Tian, Zhao Li, Yongkun Wang, Guoguang Zhang

**Affiliations:** 1 School of Electro-Mechanical Engineering, Xidian University, Xi’an 710000, China; 2 Foshan Blue Rocket Electronics Co., Ltd., Foshan 528051, China

**Keywords:** Au bump, electroplating, height uniformity, flow field, electric field

## Abstract

With the rapid development of nano/micro technology for commercial electronics, the typical interconnection method could not satisfy the high power-density packaging requirement. The 2.5D/3D integrated packaging was seen as a promising technology for nano/micro systems. The gold (Au) bump was the frequently used bonding method for these systems because of its excellent thermal, electric, and mechanical performance. However, relatively little work has been performed to analyze its height uniformity. In this study, the simulation and experimental methods were used to analyze the Au bump height uniformity. Firstly, the electroplating process of Au bump under different flow field parameters was simulated by COMSOL software. The simulated results indicated that the Au^+^ concentration polarization was the significant reason that caused the non-uniform distribution of Au bump along the wafer radius. Meanwhile, the flow field parameters, such as inlet diameter, inlet flow, titanium (Ti), wire mesh height, and Ti wire mesh density, were optimized, and their values were 20 mm, 20 L/min, 12 mm, and 50%, respectively. Subsequently, the Au bump height uniformity under different current densities was analyzed through an experimental method based on these flow field parameters. The experimental results showed that the increases of current density would decrease the Au bump height uniformity. When the current density was 0.2 A/dm^2^, the average height, range, and deviance values of Au bump were 9.04 μm, 1.33 μm, and 0.43 μm, respectively, which could reach the requirement of high density and precision for 2.5D/3D integrated packaging.

## 1. Introduction

The synergic progress of nano/micro and electronic technologies are enabling the development of miniaturization, higher efficiency, and higher power-density nano/micro electronic systems [1,2,3,4,5]. Meanwhile, these developments have brought more crucial responsibilities to electronic packaging technology. Over the past few decades, typical wire bonding technologies have been used widely in the electronic industry [6]. However, because of mismatched coefficients of thermal expansion (CTE) between the wires and chips, the interconnection areas easily occurred fatigue failure under the thermal and stress loads [7]. In addition, how to improve the quality of electronic signal transmission was also a significant challenge for wire bonding.

The 2.5D/3D Integrated packaging is seen as an effective technology to satisfy the requirements, e.g., higher input/output (I/O) density, excellent signal integrity, and outstanding heat dissipation, for nano/micro electronic systems [8,9,10,11]. The bump, through silicon via (TSV) and redistribution layer (RDL), comprises the essential components for 2.5D/3D integrated devices [12,13,14]. Among them, the functions of electric conduction, thermal conduction, and mechanical connection between TSV and RDL were realized by bumps. Thus, the reliability of devices must be assured and enhanced in terms of bumps.

Solder bumps and pure metal bumps are the two kinds of typical bumps [15,16]. The main components of solder bumps are Sn-based alloy solder [17,18,19], such as SnPb bump, SnAgCu bump, and SnBi bump, etc. However, due to the coarse shape of solder bumps, it is difficult to fabricate finely spaced bumps, which could not satisfy the requirement of high-density packaging. Furthermore, the interfacial intermetallic compounds (IMCs) growing during service and inferior electrical migration resistance of solder joints will affect the reliability and lifetime of electronic facilities [20,21,22].

Compared with the solder bumps, the pure metal bumps contain a lot of remarkable performances with electronic conductive, thermal conductive, ductility, and restriction electrical migration. Thermocompression and thermosonic are the common bonding method for pure metal bumps [23,24]. The Au bump and copper (Cu) bump are representative pure metal bumps [25,26]. Although the manufacturing price of Cu bumps is lower, the easy oxidation property of Cu is a potential threat to bonding strength because the oxide could improve bonding temperature and hinder the diffusion of Cu atoms [27,28]. Therefore, the oxidation problem should be considered when using the Cu bump.

By contrast, the Au bump obtained more attention from many laboratories and companies because of its outstanding performance, especially for oxidation resistance [29,30,31,32]. Sharma [33] et al. studied the influence of Au bump on the thermal performance of flip chip light emitting diodes (FC-LEDs) based on the Finite Element Method. Simulation results indicated that the thermal heat of FC-LEDs is directly proportional to the size of Au bump. Wang [34] et al. researched the influence of Au bump to signal integrity. The double-layer coplanar waveguide (CPW) was connected by Au-Au micro bumps and its S-parameters were tested. Experimental results showed insertion loss values < 0.07 dB at 66 GHz, which expanded the application prospect of Au bump in mm-wave frequencies signal transmission. Wu [35] et al. researched an unbiased estimator to assess and improve the Au bump manufacturing yield more accurately. Meanwhile, they pointed out a shortage of Au bump fabrication, which was usually outsourced to outsourced assembly and testing factories. Thus, researchers must make more efforts to guarantee the quality of Au bump on the forming mechanism. In addition, several studies reported the electroplating parameters influence on the bump morphology [36,37,38,39,40,41]. The related bump types and electroplating parameters were summarized in the Table 1.

The most common fabrication approaches for Au bump are the stud method and the electroplating method [42,43]. Electroplating Au bump was seen as an effective way to assemble high power density devices with simple operation and controllable size. However, few studies have reported the height uniformity of electroplating Au bump. In this study, the height uniformity meant the height difference between Au bumps on the whole wafer surface. The mathematical average height, height range, and height deviance were used together to character the Au bump height uniformity. Flow field and electric field are the crucial elements of Au bump quality in the electroplating method. Among them, four main factors of the flow field, such as inlet diameter, inlet flow, Ti wire mesh height, and Ti wire mesh density were considered, and the electric field was controlled by electric density primarily. In this study, simulative and experimental methods were used to obtain the Au bump. Meanwhile, the influences of these five factors on Au bump height uniformity were investigated, respectively.

## 2. Simulation and Experimental Methods

**Figure 1:**
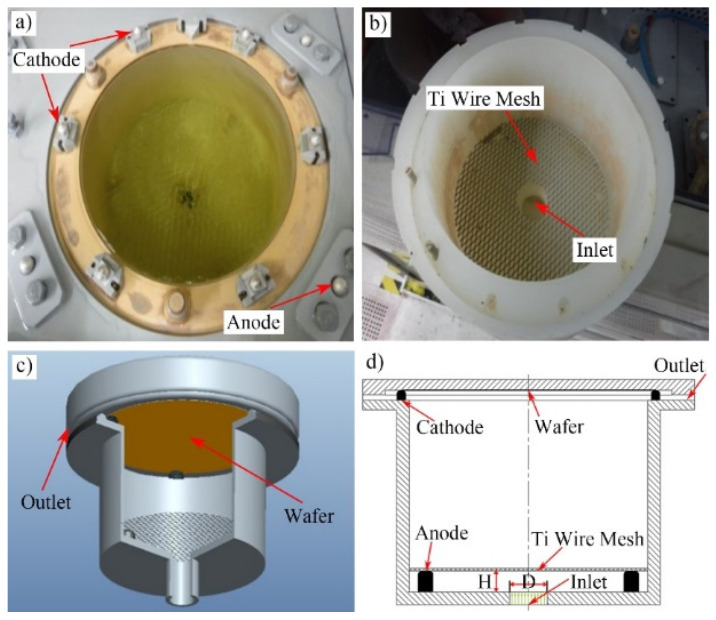
Structure of Au bump electroplating cup. (**a**) Electroplating process without wafer, (**b**) Empty electroplating cup with Ti wire mesh, (**c**) 3D model of electroplating structure with wafer, (**d**) Schematic diagram of electroplating cup.

Figure 1 shows the structure of Au bump electroplating cup. This kind of electroplating cup could be found in the most of common commercial electroplating equipment. In the process of electroplating Au bump, the wafer was connected with cathodes firstly, and the positions and morphologies of cathodes are shown in Figure 1a. Subsequently, the electroplating bath would enter the inlet and across the Titanium (Ti) alloy wire mesh, and their positions and morphologies are shown in Figure 1b. The Ti wire mesh was connected with anodes. Then, the electroplating bath was sprayed to the wafer surface and deposited Au bump through electrochemical action. Finally, the electroplating bath would outflow from the outlet. The 3D model of the whole electroplating structure is shown in Figure 1c. Figure 1d shows the 2D schematic diagram of electroplating cup, and H and D represent the height of Ti wire mesh and the diameter of inlet, respectively.

Metal aluminum (Al) was always selected as the I/O electrode of chips to reduce the fabrication cost. However, due to the disadvantage of Al with easy corrosiveness and difficult connection to Au, the under bump metallurgy (UBM) layer was sputtered or evaporated on the Al electrode as a transition layer. Meanwhile, the Au is the preferred material for the outermost layer to prevent the oxidation of UBM, and the thickness of Au layer was less than 100 nm usually. During the electroplating process, this Au layer connected with the cathode directly and its thickness improved as time increased. Therefore, this thin Au layer was assumed as a shell electrode, which was the electroplating cathode in the flow field simulation. In the electroplating experiment, the UBM layer was composed of the Au layer and TiW layer. Among them, the Au layer with 85 nm acted as the antioxidation and seed layer, the TiW layer with 350 nm between the Au layer, and wafer played the role of adhesion and barrier layer.

**Figure 2:**
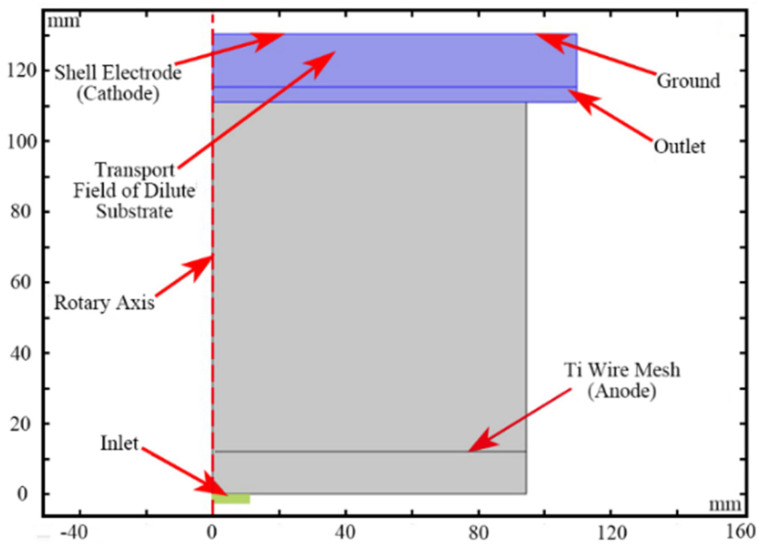
2D rotation cross-section of flow field simulation model.

In order to enhance the flow field simulation efficiency and speed, other assumptions were made as follows: (1) Au bumps were distributed throughout the whole wafer without clearance; (2) the whole electroplating area was symmetrically distributed; (3) the two-dimensional (2D) rotation model could replace the 3D electroplating area perfectly and (4) the Ti wire mesh was set to an anode. Boundary conditions were set according to the real craft. The 2D rotation cross-section of the flow field simulation model was shown in Figure 2, the red dotted line represented the rotary axis, and the transport field of dilute substrate was used to simulate the transmission of Au+ near the wafer surface.

Considering the practical flow of electroplating bath during electroplating, the turbulence shear stress transport (TSST) model was established. In the actual craft of Au bump manufacture, four significant factors, such as inlet diameter, inlet flow, Ti wire mesh height, and Ti wire mesh density, controlled the distribution of electroplating bath. Among them, the Ti wire mesh density was expressed as a percentage of the Ti metal area to total area. Thus, different parameters of these four factors were set to simulate the fluid flow in the electroplating cup. The COMSOL Multiphysics software was used to simulate the electroplating process and obtain the height of Au bump based on the different flow fields. Table 2 shows the related simulation parameters in the flow field simulation.

In addition, the analysis of electric field mainly considered current densities, and the experimental method was adopted to acquire the Au bump under different current densities. The common commercial fully automatic spraying equipment was used to complete the electroplating Au bump experiment, and the height of Au bump was measured by the wafer probe measurement platform. The morphology of Au bump was observed by the metallographic microscope and scanning electron microscope (SEM).

## 3. Results and Discussion

### 3.1. Influence of Flow Field

**Figure 3:**
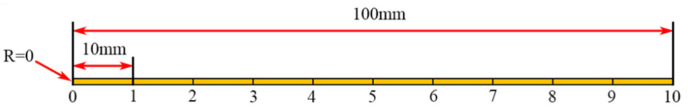
Schematic diagram of Au bump height measurement method.

As mentioned above, the inlet diameter, inlet flow, Ti wire mesh height, and Ti wire mesh density were four significant factors of the flow field. To analyze the influence of different factors on the Au bump uniformity, the measurement method was adopted to acquire the height of Au bump, as shown in Figure 3. In practical production, the wafer diameter was eight inches. Thus, according to the 2D flow field simulation model in Figure 2, the linear measurement method was selected as shown in Figure 3. The measurement points were spaced 10 mm uniformly and eleven points were obtained from the rotary axis to the edge of 2D simulation model.

In order to analyze the Au bump height uniformity, the range (*R*) and deviance (*D*) were selected to illustrate the uniformity of Au bump height. The calculation of them can be expressed in Equations (1) and (2):

(1) $$R=h_{\max}-h_{\min}$$

(2) $$D=\frac{1}{n}\cdot {\left(\sum_{i=1}^{n}\left(h_{i}-h_{\mathrm{avg}}\right)\right)}^{\frac{1}{2}}$$

Here, *h*_max_, *h*_min_, and *h*_avg_ are the maximum height, minimum height, and average height of Au bump in measurement points, respectively.

#### 3.1.1. Influence of Inlet Diameter

In this section, the inlet diameters were set to 12 mm, 14 mm, 16 mm, 18 mm, and 20 mm, respectively. In addition, the inlet flow, Ti wire mesh height, and Ti wire mesh density were 26 L/min, 12 mm, and 27%, severally. The simulation time, which is also the electroplating time, was set to 30 min.

**Figure 4:**
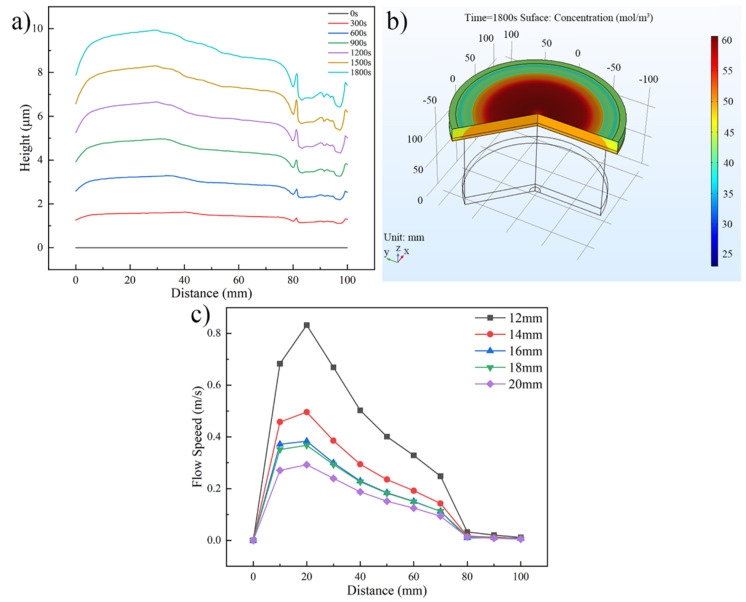
Distributions of the Au bump height, Au+ concentration, and flow speed. (**a**) Au bump height distribution along the wafer radius (inlet diameter is 12 mm), (**b**) Au+ concentration distribution on the wafer surface (inlet diameter is 12 mm), and (**c**) flow speed distribution on the wafer surface at different inlet diameters.

The distributions of the Au bump height, Au+ concentration and flow speed under different inlet diameters are shown in Figure 4. Figure 4a shows the distribution of Au bump height along the wafer radius when the inlet diameter is 12 mm. As shown in this figure, the Au bump height increases from 7.874 μm to 9.929 μm when the wafer radius improves from 0 mm to 30 mm. With further extension of the wafer radius, the Au bump height shows a downtrend, and the lower Au bump appears between 80 mm to 100 mm of the wafer radius. The difference of Au+ concentration is seen as the significant reason which causes the Au bump height changes along the wafer radius. Figure 4b shows the Au+ concentration on the wafer surface when the inlet diameter is 12 mm. Within the wafer radius of 10 mm to 50 mm, the Au+ concentration with 60 mol/m3 near the wafer surface is greater than that of the electroplating bath. Compared with this, Au+ concentration at the edge of the wafer is lower than that of the electroplating bath. The changes of Au+ concentration are caused by the flow speed on the wafer surface. The flow speed distribution on the wafer surface at different inlet diameters is shown in Figure 4c. The flow speed exceeds 0.4 m/s at the wafer radius of 10 mm to 50 mm. The stronger convection improves the Au+ concentration, thus the higher Au bump occurs in this area. In contrast, the slower flow speed within the wafer radius of 80 mm to 100 mm decreases the Au+ concentration and Au bump height.

**Figure 5:**
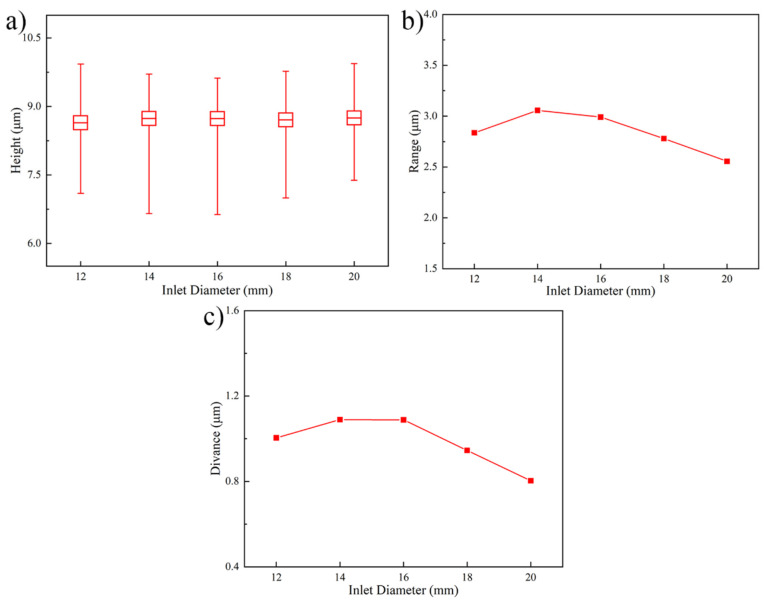
Au bump height and uniformity under different inlet diameters. (**a**) Au bump height, (**b**) Au bump range, and (**c**) Au bump deviance.

Figure 5 shows the Au bump height and uniformity under different inlet diameters. As shown in Figure 5a, the Au bump average height does not express prominent variation in statistics, as the increase of inlet diameter and its value is about 8.70 ± 0.05 μm. The range and deviance of Au bump in Figure 5b,c show the downtrend generally as the inlet diameter improves. When the inlet diameter improves from 12 mm to 20 mm, the height range decreases from 2.836 μm to 2.556 μm and the height deviance declines from 1.003 μm to 0.803 μm. The improvement of Au bump uniformity is due to the change of flow speed. As the inlet diameter ranges from 12 mm to 20 mm, the maximum flow speed descends from 0.832 m/s to 0.293 m/s and the distribution of flow field would become more uniform, as shown in Figure 4c. According to the Figure 4 discussion above, the stronger flow speed would generate Au+ concentration polarization, which caused the larger height difference between the wafer center and edge. The slower flow speed and uniform flow field would weaken the Au+ concentration polarization. Thus, the enhancement of inlet diameter has a positive effect on the height uniformity of Au bump.

#### 3.1.2. Influence of Inlet Flow

As discussed in Section 3.1.1, the inlet diameter with 20 mm possessed the optimal Au bump uniformity. Therefore, in the simulation of inlet flow, the inlet diameter was set as 20 mm, and the inlet flows were set as 20 L/min, 26 L/min, 30 L/min, 35 L/min, and 40 L/min, respectively. The other simulation parameters were the same as before.

**Figure 6:**
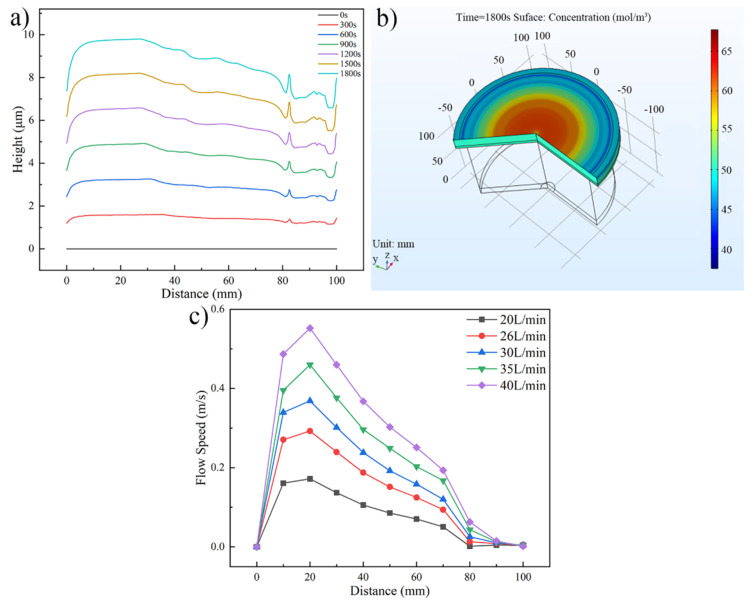
Distributions of the Au bump height, Au+ concentration and flow speed. (**a**) Au bump height distribution along the wafer radius (inlet flow is 40 L/min), (**b**) Au+ concentration distribution on the wafer surface (inlet flow is 40 L/min), and (**c**) flow speed distribution on the wafer surface at different inlet flows.

Figure 6 shows the distributions of the Au bump height, Au+ concentration, and flow speed under different inlet flows. Figure 6a shows the Au bump height along the wafer radius, as the inlet flow is 40 L/min. The distribution trend is similar with Figure 5a. In the above discussion, the concentration polarization of Au+ was caused by different flow speeds on the wafer surface, thus the Au bump height appears different along the wafer radius. The Au+ distribution on the wafer surface in Figure 6b exhibits the concentration polarization phenomenon caused by the difference of flow speed when the inlet flow is 40 L/min. The flow speed along the wafer surface under different inlet flows is shown in Figure 6c. In this figure, when the inlet flow increases from 20 L/min to 40 L/min, the maximum value of flow speed improves from 0.172 m/s to 0.552 m/s. Meanwhile, the distribution of flow speed becomes more uneven.

**Figure 7:**
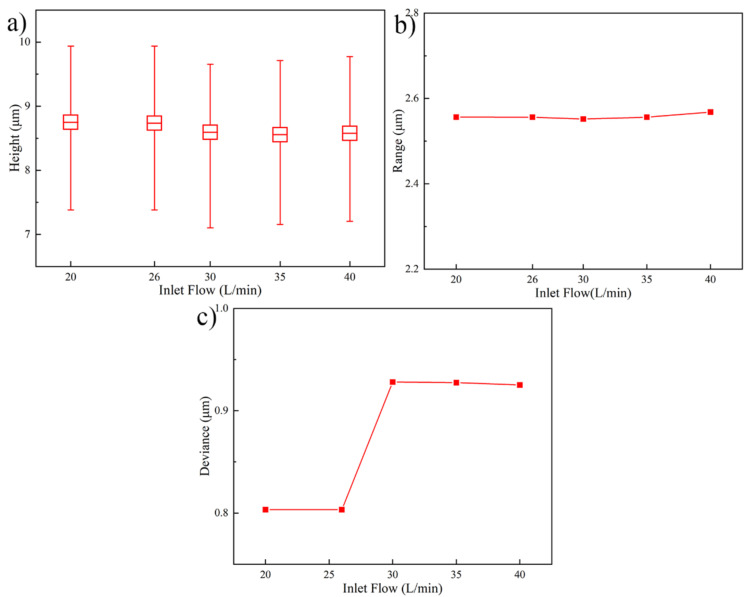
Au bump height and uniformity under different inlet flows. (**a**) Au bump height, (**b**) Au bump range, and (**c**) Au bump deviance.

Figure 7 shows the influence of different inlet flows on the Au bump uniformity. As shown in Figure 7a,b, the alter of inlet flow has limited influence on the Au bump height and range. As the inlet flow enhances from 20 L/min to 40 L/min, the values of Au bump height and range are 8.65 ± 0.10 μm and 2.55 ± 0.05 μm, respectively. In contrast, the Au bump height deviance in Figure 7c possesses volatility with the increase of inlet flow. The main reason caused this phenomenon is the difference of flow fields under different inlet flows. The deviance value of Au bump height improves from 0.803 μm to 0.920 μm when the inlet flow ranges from 20 L/min to 30 L/min. Then, the Au bump height deviance value increases slowly by magnifying the inlet flow.

#### 3.1.3. Influence of Ti Wire Mesh Height

According to the analysis in Section 3.1.1 and Section 3.1.2, when the inlet diameter and flow are 20 mm and 20 L/min, respectively, a better Au bump height uniformity could be acquired. Thus, in the simulation of Ti wire mesh height, their parameters were selected. Moreover, the Ti wire mesh heights were selected as 12 mm, 20 mm, 30 mm, 40 mm, 50 mm, and 80 mm, severally. The Ti wire mesh density was 27%, as mentioned above.

**Figure 8:**
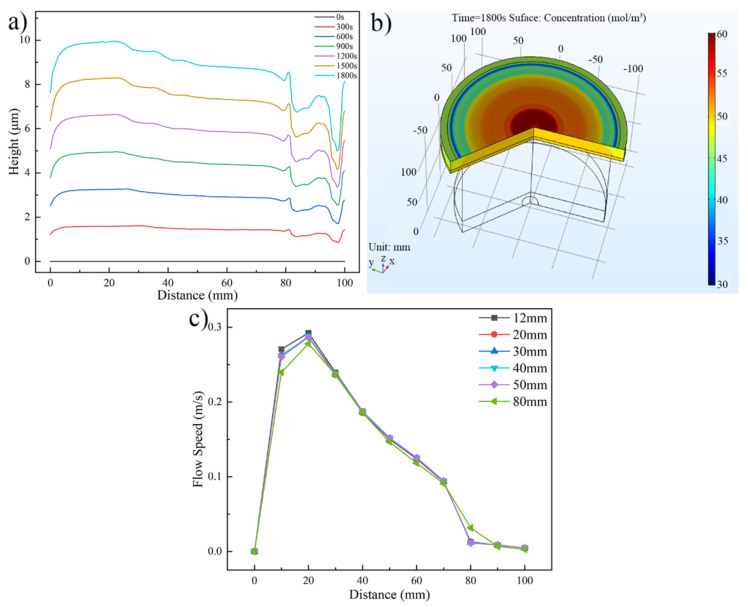
Distributions of the Au bump height, Au+ concentration, and flow speed. (**a**) Au bump height distribution along the wafer radius (Ti wire mesh height is 12 mm), (**b**) Au+ concentration distribution on the wafer surface (Ti wire mesh height is 12 mm), and (**c**) flow speed distribution on the wafer surface at different Ti wire mesh heights.

Figure 8 shows the distributions of the Au bump height, Au+ concentration, and flow speed under different Ti wire mesh heights. The Au bump height and Au+ concentration distribution along the wafer radius are shown in Figure 8a,b, respectively. In these figures, the distribution of Au bump height and Au+ concentration has satisfactory consistency, as discussed in the previous two sections. Figure 8c is the flow speed distribution on the wafer surface at different Ti wire mesh heights. As shown in this figure, the restricted variety of flow speed occurs with the changes of Ti wire mesh height. When the Ti wire mesh height increases from 12 mm to 80 mm, the maximum value of flow speed decreases from 0.293 m/s to 0.278 m/s, which fell by only 5.1%.

**Figure 9:**
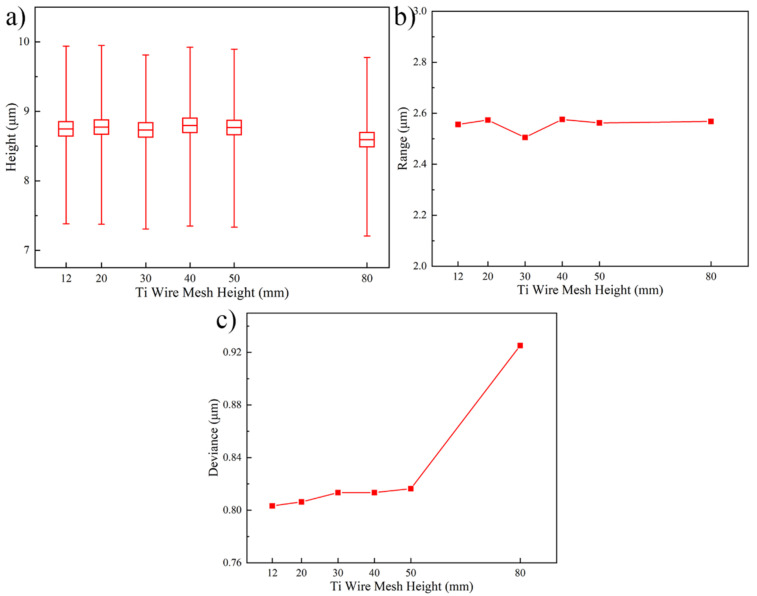
Au bump height and uniformity under different Ti wire mesh heights. (**a**) Au bump height, (**b**) Au bump range, and (**c**) Au bump deviance.

Figure 9 shows the Au bump height and uniformity under different Ti wire mesh heights. As shown in Figure 9a,b, the Au bump height and range are 8.70 ± 0.10 μm and 2.53 ± 0.03 μm, respectively. The non-obvious vibration of their value indicates that the limited influence occurs when the Ti wire mesh height increases from 12 mm to 80 mm. However, the deviance of Au bump height in Figure 9c shows a rising trend with the enhancement of Ti wire mesh height. The deviance value of Au bump height improves from 0.803 μm to 0.816 μm as the Ti wire mesh height enhances from 12 mm to 50 mm. Further rising the Ti wire mesh height to 80 mm, this value reaches 0.925 μm. In the electroplating process, the electroplating bath enters the cup from the inlet and then through the Ti wire mesh. Here, the Ti wire mesh is a rectifier for the whole electroplating model. Thus, the lower height of Ti wire mesh could rectify the electroplating bath preferably and the flow field would be more uniform in this situation. This phenomenon suggests that the opportune reduction of Ti wire mesh height is beneficial to enhance the Au bump height uniformity.

#### 3.1.4. Influence of Ti Wire Mesh Density

As described in the three sections above, the Au bump possessing the preferable height uniformity as inlet diameter, inlet flow, and Ti wire mesh height are 20 mm, 20 L/min, and 12 mm, respectively. In order to obtain a more uniform Au bump, these parameters were chosen in this section and the Ti wire mesh densities were set to 5%, 10%, 20%, 27%, 40%, and 50%.

**Figure 10:**
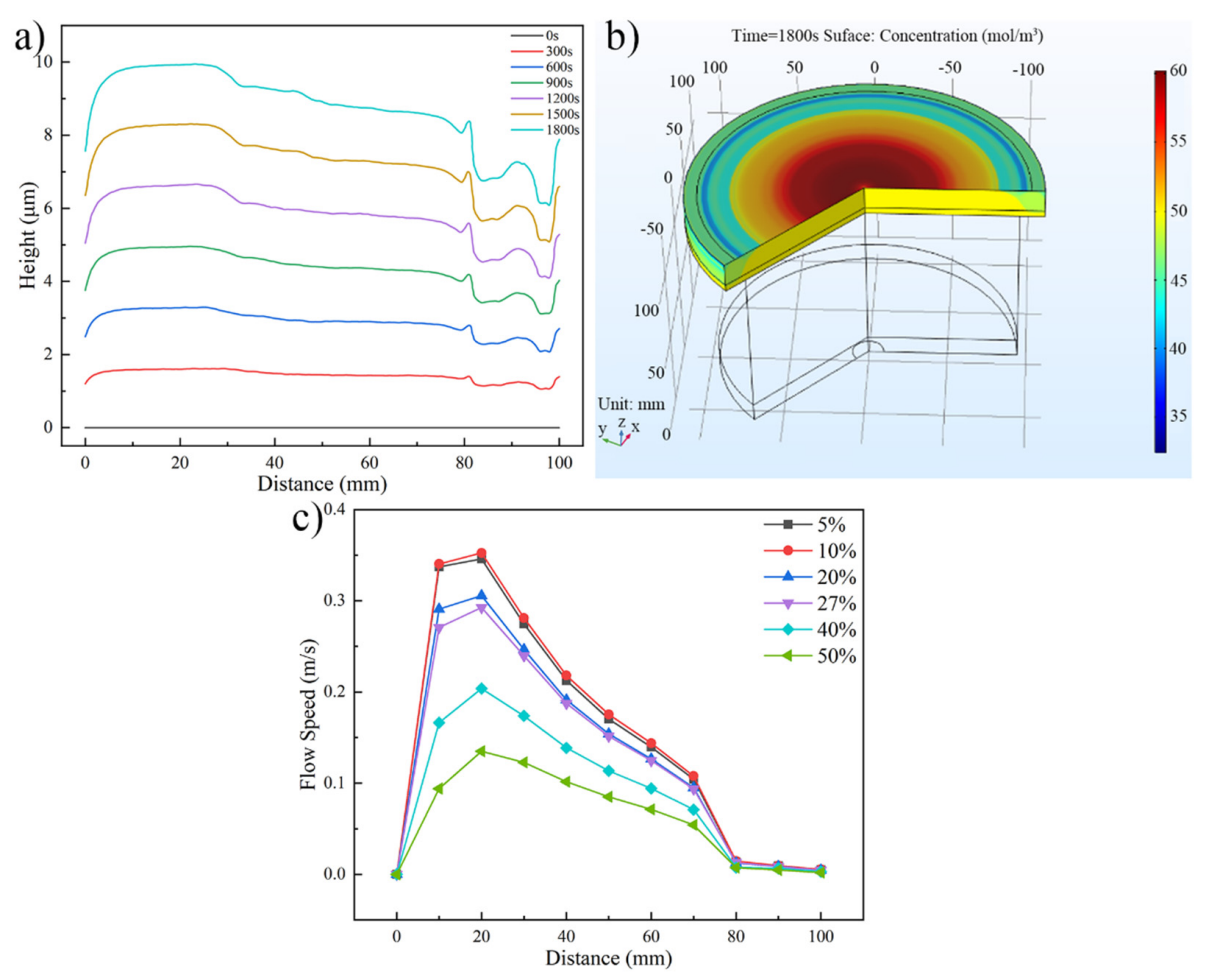
Distributions of the Au bump height, Au+ concentration, and flow speed. (**a**) Au bump height distribution along the wafer radius (Ti wire mesh density is 5%), (**b**) Au+ concentration distribution on the wafer surface (Ti wire mesh density is 5%), and (**c**) flow speed distribution on the wafer surface at different Ti wire mesh densities.

Figure 10 shows the distributions of Au bump height, Au+ concentration, and flow speed under different Ti wire mesh densities. As shown in Figure 10a,b, the distributions of Au bump height and Au+ concentration along the wafer radius are similar to the previous three sections. Furthermore, according to Figure 4a, Figure 6a, Figure 8a and Figure 10a, the poor height uniformity of Au bump occurs as the electroplating time improves. Figure 10c shows the flow speed distribution on the wafer surface at different Ti wire mesh densities. In this figure, the flow speed shows a downtrend as the improves of Ti wire mesh density. The maximum flow speed decreases from 0.346 m/s to 0.135 m/s as the Ti wire mesh density increases from 5% to 50%. It suggests that the rectifying effect of Ti wire mesh on electroplating bath would become better, and the flow field is more uniform with the increases of Ti wire mesh density.

**Figure 11:**
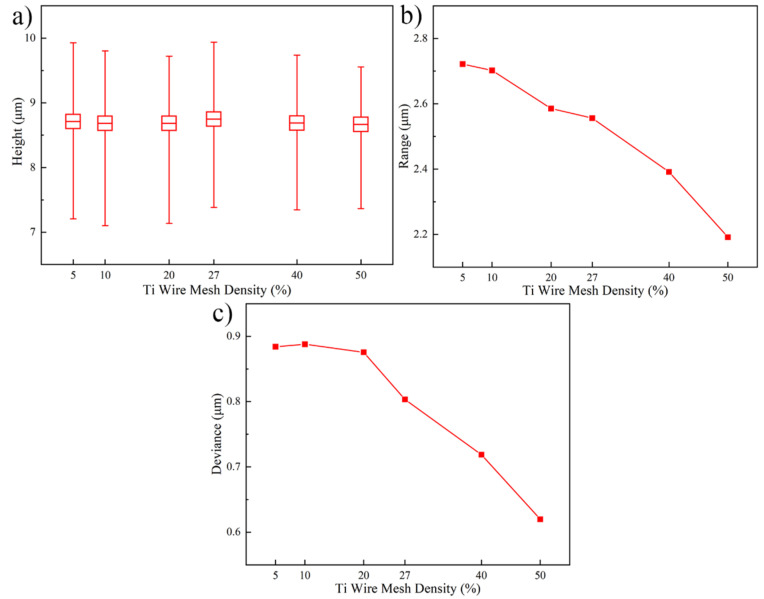
Au bump height and uniformity under different Ti wire mesh densities. (**a**) Au bump height, (**b**) Au bump range, and (**c**) Au bump deviance.

Figure 11 shows the influence of different Ti wire mesh densities on the Au bump height and uniformity. The Au bump height with 8.70 ± 0.02 μm is shown in Figure 11a. The changes of Ti wire mesh density have a finite effect on the Au bump height. Figure 11b,c show the Au bump height range and deviance, respectively. As shown in these figures, the height range and deviance of Au bump decrease with the increases of Ti wire mesh density. When the Ti wire mesh density improves from 5% and 27%, the value of height range descends from 2.722 μm to 2.192 μm and the height deviance value declines from 0.884 μm to 0.620 μm. As mentioned before, the Ti wire mesh is a rectifier for the electroplating model. As other parameters were fixed, the higher density of Ti wire mesh would enhance the uniformity of flow field. Thus, the uniformity of Au bump height improves as the density of Ti wire mesh increases.

### 3.2. Influence of Electric Field

In this section, the influences of different current densities on the Au bump height uniformity were analyzed through an experiment. The electroplating flow field parameters were optimized through simulation in Section 3.1 and used in the experiment. Here, the inlet diameter, inlet flow, Ti wire mesh height, and Ti wire mesh density were set as 20 mm, 20 L/min, 12 mm, and 50%. In addition, the current densities were selected to 0.2 A/dm^2^, 0.3 A/dm^2^, 0.4 A/dm^2^, 0.5 A/dm^2^, 0.6 A/dm^2^, and 0.8 A/dm^2^, respectively.

**Figure 12:**
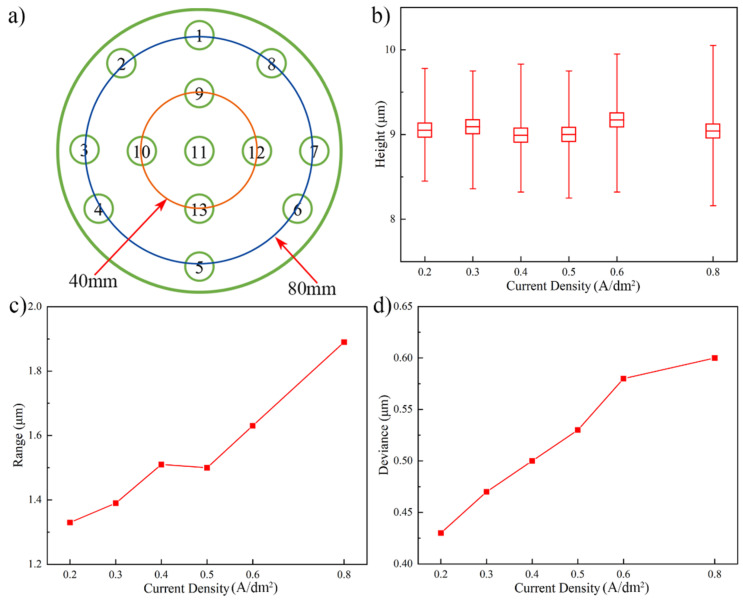
Influence of current density on the Au bump height uniformity in experiment. (**a**) Schematic diagram of test points distribution on the wafer, (**b**) Au bump height, (**c**) Au bump range, and (**d**) Au bump deviance.

Figure 12 shows the experimental results of different current densities influences on the Au bump height and uniformity. In order to obtain the Au bump height on the surface of wafer, thirteen test points were chosen, and the distribution diagram of them is shown in Figure 12a. The Au bump height with 8.95 ± 0.10 μm in Figure 12b indicates that the limited influence occurs on the Au bump height as the changes of current density. Figure 12c,d show the height range and deviance under different current densities in the experiment. In these figures, the height range and deviance enhance from 1.32 μm and 0.43 μm to 1.88 μm and 0.60 μm, with the current density increasing from 0.2 A/dm^2^ to 0.8 A/dm^2^, respectively. The experiment result demonstrates that the Au bump height uniformity would enhance with the decreases of current density. In addition, the smaller values of Au bump height range and deviance indicate that the simulation is an effective method to optimize the electroplating process.

**Figure 13:**
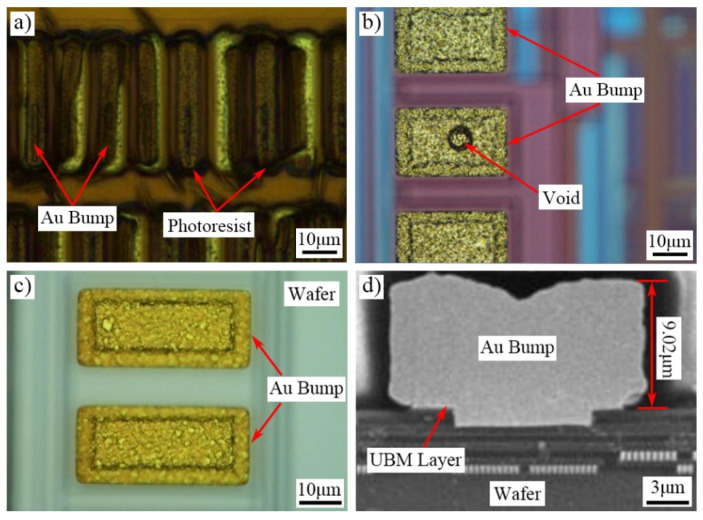
Morphology of Au bump. (**a**) Photoresist burning, (**b**) Void, (**c**) Top view of optimized Au bump, (**d**) Cross-section view of optimized Au bump.

Figure 13 shows the morphology of Au bump fabricated in the experiment. Figure 13a,b show the photoresist burning and void defects during the Au bump fabrication, respectively. The top view of optimized Au bump is shown in Figure 13c. In this figure, the length and width of Au bump are 40 μm and 20 μm, respectively. In addition, the space between two Au bumps is 10 μm. Meanwhile, obvious voids or gaps could not be observed on the Au bump surface. Thus, the high-quality Au bump with satisfying uniformity can be obtained through experiment. Figure 13d shows the SEM cross-section view of Au bump. The Au bump with 9.02 μm height is electroplated on the wafer. The bright line between Au bump and wafer represents the UBM layer. The Au bump appears saddle-shaped, which means low in the middle and high on all sides. According to the study of Li et al. [44], the current crowding effect was seen as the significant reason caused this profile. Luo et al. [45] proposed active-area density model to explain the current crowding effect in their study. According to this model, regions that are more densely populated with photoresist tend to attract a high current density, and hence the current crowding effect would appear. As the electroplating is proportional to the current density, a thicker film is deposited on the edge of a wide structure due to the increased current density, whereas the middle of the structure is thinner. They also found that the current crowding should always exist for patterned wafer plating. The study of Wu [35] et al. also demonstrated the existence of this Au bump morphology.

**Figure 14:**
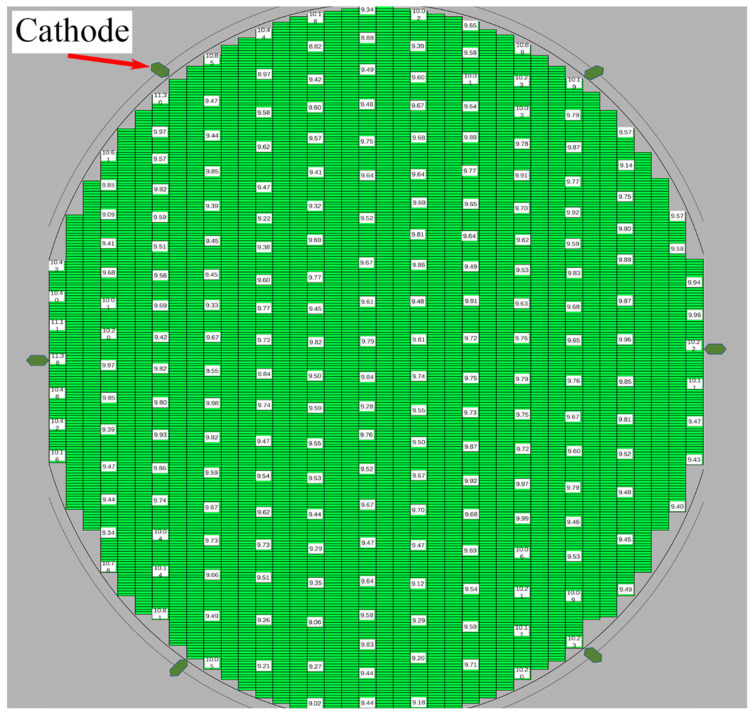
Au bump distribution on the whole wafer surface.

Figure 14 shows the Au bumps distribution on the whole wafer surface. As shown in the figure, the total numbers of Au bumps are 216. The maximum height value of Au bump is 11.38 μm, and the minimum height value is 8.82 μm. To intuitively investigate Au bump height variation on the whole wafer surface, the statistical data of Au bump height is shown in Table 3. In this table, the total number of Au bumps in the 8~9 μm and 11~12 μm height interval is 6, which only accounts for 2.78% of all Au bumps. It is meaningless to analyze Au bump height uniformity with fewer height samples. Thus, the height uniformity of these Au bumps was ignored. In contrast, 83.33% Au bumps were at the 9~10 μm height interval. The number, average height, height range, and height deviance of these Au bumps are 180, 9.61 μm, 0.97 μm, and 0.20 μm, respectively. Meanwhile, there are 13.89% Au bumps at the 9~10 μm height interval, The number, average height, height range, and height deviance of them are 30, 10.28 μm, 0.87 μm, and 0.26 μm, respectively.

## 4. Conclusions

In this study, the simulation and experimental methods were used to fabricate the Au bump and analyze its height uniformity. Four significant flow field parameters, with inlet diameter, inlet flow, titanium (Ti) wire mesh height, and Ti wire mesh density, were optimized by the COMSOL software, and their values were 20 mm, 20 L/min, 12 mm, and 50%, respectively. The simulation results indicated that the Au^+^ concentration polarization was considered as the main reason that caused the non-uniform distribution of Au bump height along the wafer radius. The inlet diameter and Ti wire mesh density were positively associated with the Au bump height uniformity. In contrast, the decrease of inlet flow and Ti wire mesh height would improve the height uniformity of Au bump. Based on the optimized flow field parameters, the different current densities were set to fabricate the Au bump during the experimental method. The results indicated that the increases of current density would decrease the Au bump height uniformity. The high-precision Au bump with 1.33 μm height range and 0.43 μm height deviance was obtained when the current density was 0.2 A/dm^2^. Thus, the optimized flow field parameters by simulation were effective in fabricating the high-quality Au bump. In addition, the changes of flow field and electric field parameters had limited influence on the Au bump height.

## Figures and Tables

**Table 1:** Bump types and electroplating parameters.

Bump Types	Electroplating Parameters		
	Current Density	Electroplating Time	Electroplating Temperature
Au-Sn [36]	0.5 A/dm^2^	60 min	50 °C
	1 A/dm^2^	13 min	50 °C
Sn-Cu [37]	1–8 A/dm^2^	2 h	-
Sn-Pb [38]	6 A/dm^2^	3 h	-
Sn-Ag [38]	6 A/dm^2^	1 h	-
Sn-Bi [39]	10–30 mA/cm^2^	-	Room temperature
In [40]	0.2 mA/cm^2^	20 min	Room temperature
Cu [41]	6 A/dm^2^	5 min	-

**Table 2:** Flow field simulation parameters of electroplating Au bump.

Name	Symbol	Value	Unit
Au^+^ concentration in electroplating bath	*C_0_*	50	mol/m^3^
Molar mass of deposition (Au)	*M*	197	g/mol
Conductivity of cathode (Au)	*σ_1_*	4.52	S/m
Thickness of cathode (Au)	*S*	8.5 × 10^−9^	m
Conductivity of electroplating bath	*σ_2_*	37.5	S/m
Density of electroplating bath	*ρ*	1100	kg/m^3^
Temperature of electroplating bath	*T*	323	K
Kinematic viscosity of electroplating bath	*μ*	0.001	Pa·s
Initial electric potential of anode	*Phil*	2	V
Equilibrium electric potential	*E_eq_*	1.69	V

**Table 3:** Au bump height statistical data.

height Interval (μm)	8~9	9~10	10~11	11~12
numbers	3	180	30	3
average (μm)	-	9.61	10.28	-
range (μm)	-	0.97	0.87	-
deviance (μm)	-	0.20	0.26	-

## Data Availability

The data presented in this study are available on request from the corresponding author.
